# Clinical characteristics, clinical laboratories, and risk factors of elderly patients with chronic obstructive pulmonary disease complicated with pulmonary embolism

**DOI:** 10.3389/fmed.2025.1421169

**Published:** 2025-05-14

**Authors:** Chunhua Jin, Yanping Hu, Fang Liu

**Affiliations:** ^1^Emergency Department of Shaoxing People’s Hospital, Shaoxing, Zhejiang, China; ^2^Emergency Department, Dalian University Affiliated Xinhua Hospital, Dalian, Liaoning, China

**Keywords:** chronic obstructive pulmonary disease, pulmonary embolism, red cell distribution width, clinical characteristics, risk factors

## Abstract

**Purpose:**

The objective of this research is to examine the occurrence, clinical manifestations, and determinants of pulmonary embolism (PE) in older individuals diagnosed with chronic obstructive pulmonary disease (COPD).

**Methods:**

A retrospective analysis was performed on elderly patients diagnosed with COPD, who were admitted to five different hospitals within our province. These patients were categorized into two groups based on the presence or absence of pulmonary embolism (PE). And systematically compile and examine the foundational data, clinical attributes, and pertinent laboratory parameters outlined in their respective medical records. These encompass blood routine, arterial blood gas analysis, coagulation markers, and biochemical indicators.

**Results:**

A total of 958 elderly patients with COPD were included in the study. Among them, 121 patients (12.63%) were found to have complications with PE. During hospitalization, 50 cases (5.22%) resulted in death. In the multivariate analysis, several factors were found to be significantly associated with a higher prevalence of PE, including age, female gender, deep vein thrombosis, hypertension, PaCO_2_ ≤ 40 mmHg, and normal clinical signs and symptoms on chest X-rays (*p* < 0.05). The RDW-SD and RDW-CV values of the COPD combined with the PE group were significantly higher compared to those of the COPD without PE group (*p* < 0.001). The risk of PE caused by higher RDW-SD was significantly greater than that caused by lower RDW-SD (*p* < 0.05). The area under the curve for RDW-SD in predicting PE is 0.723. The critical value of RDW-SD was determined to be 46.25, with a sensitivity of 75.59% and a specificity of 67.5%.

**Conclusion:**

It is essential to give careful attention to the prevalence and factors that put elderly patients with COPD at risk for PE. The utilization of RDW could potentially serve as a predictive tool for identifying the onset of PE in COPD patients.

## 1 Introduction

Pulmonary thromboembolism (PTE), primarily manifested as pulmonary embolism (PE) in 90% of cases (1, 2), occurs when thrombi occlude pulmonary vessels. Although dual pulmonary circulation usually prevents pulmonary infarction (3), deep venous thrombosis (DVT) remains the predominant embolic source (4, 5). COPD exacerbates PE risk through inflammation-mediated endothelial dysfunction and hypercoagulability (6), with clinical presentations often mimicking acute COPD exacerbations, leading to frequent misdiagnosis (7–9). Timely diagnosis critically impacts prognosis, requiring clinician expertise in selecting appropriate diagnostic methods (10), where CTPA and laboratory indicators prove particularly valuable (11).

CTPA has become the primary diagnostic modality for PE (12), offering non-invasive 3D vascular reconstruction with high spatial resolution (13). Its technical advantages include rapid subsegmental emboli detection and reduced respiratory artifacts (14), maintaining high sensitivity/specificity while ensuring cost-effectiveness (15).

COPD’s chronic hypoxia induces erythrocytosis and impairs coagulation factor clearance (16), while systemic inflammation activates coagulation pathways through endothelial damage (17). Although inflammatory factors may drive coagulation changes as extrapulmonary COPD manifestations (18), conventional indicators (APTT, PT) reflect endogenous pathway activation (19), with D-dimer indicating coagulation-fibrinolysis interplay (20). Notably, PaCO2 levels show significant decreases in COPD-PE patients (21). Over the past few years, a rise has been observed in the frequency of Red Cell Distribution Width (RDW) in a wide range of illnesses. This observation implies that RDW holds the potential as a valuable marker for distinguishing between different diseases (22).

Coagulation function and arterial blood gas analysis have established reference values for diagnosing COPD combined with PE. However, currently, CTPA examination remains one of the most reliable, fastest, and sensitive methods for diagnosing PE. Coagulation function and blood gas analysis provide clues for diagnosing COPD combined with PE and also have value in preventing the occurrence of this combination. This research is to retrospectively analyze elderly patients with COPD and PE, exploring the risk factors and predictive value of clinical characteristics, CTPA results, and laboratory indicators.

## 2 Methods

### 2.1 Research object

This study adhered to the case inclusion criteria and analyzed 958 elderly patients diagnosed with COPD from 5 different hospitals in our province between June 1, 2022, and May 31, 2023. A retrospective study was carried out by categorizing elderly patients with COPD into two groups according to the occurrence of PE. The initial cohort, denoted as the observation group, was composed of PE-inflicted elderly COPD patients. In contrast, the control group included elderly COPD patients without PE. The study was sanctioned by the institutional review board at each participating center and adhered to the principles outlined in the Declaration of Helsinki. Considering its retrospective design, the necessity for written informed consent was waived.

### 2.2 Inclusion and exclusion criteria

Inclusion criteria: (1) The age of the patient is ≥60 years old; (2) According to the global initiative on chronic obstructive pulmonary disease (GOLD) standard (23), doctors diagnosed patients with COPD. Additionally, patients who were previously hospitalized and diagnosed with COPD at discharge, as well as those receiving treatment for COPD, were included; (3) The patient was diagnosed with PE based on the criteria outlined in the ESC Pulmonary Embolism Diagnosis and Treatment Guidelines (24).

Exclusion criteria: (1) Individuals with prior blood transfusion records and present hematologic disorders (e.g., anemia, irregular blood cell production, and blood cancers); (2) Participants lacking complete or accessible data.

### 2.3 Data collection

Basic information, like age and gender, should be included in the medical records of older individuals. Additionally, clinical features including blood pressure, pulse rate, and signs and symptoms associated with deep vein thrombosis (DVT), like pain, redness, and swelling of the affected limb, must be documented. Electrocardiogram attributes such as right bundle branch, right QRS axis deviation, and S1Q3T3 should also be noted, in addition to radiological findings from chest X-rays and any other pertinent information.

Laboratory parameters including complete blood count (CBC), arterial blood gas (ABG) analysis, coagulation profile, and biochemical indices were systematically collected. The CBC evaluation encompassed: white blood cell (WBC) count, red blood cell (RBC) count, hemoglobin (Hb) concentration, platelet count (PLT), red cell distribution width (RDW) parameters [coefficient of variation (RDW-CV) and standard deviation (RDW-SD)], mean platelet volume (MPV), platelet distribution width (PDW), and hematocrit (Hct). RDW-CV was calculated as (standard deviation of erythrocyte volume/mean corpuscular volume [MCV]) × 100, reflecting relative volumetric variation, while RDW-SD provided absolute erythrocyte size distribution in femtoliters (fL). Comorbid conditions (e.g., heat exhaustion, diabetes mellitus) and established PE risk factors including malignancy and prior venous thromboembolism were concurrently documented.

### 2.4 Statistical method

The IBM SPSS 26.0 software was employed to analyze all the data in this particular study. To assess the normal distribution of the dataset, the Shapiro–Wilk method was implemented, and any observed differences were statistically considered significant if *p* < 0.05. For continuous variables, the mean ± standard deviation (SD) or median values were used to present the data, and the Student t-test or Mann–Whitney U test was utilized for analysis. As for categorical data, cases (counts) or percentages were presented, and the chi-square test was employed for analysis. Relevant variables were included in the logistic regression model. This model aimed to determine the independent risk factors associated with COPD patients who had PE. The presence of PE was also evaluated in terms of its impact on in-hospital mortality and length of stay. To assess the discriminant performance of the parameters, the receiver operating characteristic (ROC) curve was utilized. The corresponding area under the curve (AUC) with a 95% confidence interval (CI) was employed for comparing predicted probabilities. The optimal cutoff value, determined based on the Youden index, was used to calculate the sensitivity and specificity cutoff values of the parameters.

## 3 Results

### 3.1 Analysis of baseline demographic characteristics of patients and potential clinical characteristics and risk factors of PE

**Figure 1:**
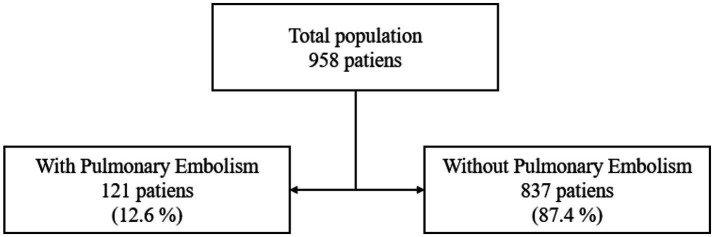
Grouping information.

Among the 958 elderly patients diagnosed with COPD, with a mean age of 75.4 ± 7.6 years and 65.6% being male, a total of 121 patients were diagnosed with PE. This corresponds to an average prevalence of 12.63% (Figure 1). Table 1 provides a list of baseline demographics, potential clinical characteristics, and risk factors associated with PE.

**Table 1:** Comparison of baseline characteristics and clinical complications of included patients.

	Total population		COPD with PE		COPD without PE		*t/X^2^*	*p*-value
	*n*	%	*n*	%	*n*	%		
*N*	958	100.0	121	100.0	837	100.0		
Age (Mean ± SD, years)	75.4 (7.6)		78.6 (7.7)		74.9 (7.4)		5.08	<0.001
Sex - Men	628	65.6	61	50.4	567	67.7	14.06	<0.001
Recent bed rest ≥3 days	238	24.8	41	33.9	197	23.5	6.06	0.01
Recent surgery	32	3.3	7	5.8	25	3.0	2.56	0.11
Previous venous thromboembolism	86	9.0	20	16.5	66	7.9	9.66	0.002
Malignant tumor	192	20.0	17	14.0	175	20.9	3.10	0.08
Heart failure	104	10.9	17	14.0	87	10.4	1.46	0.23
Diabetes Mellitus	158	16.5	16	13.2	142	17.0	1.08	0.30
Hypertension	247	25.8	41	33.9	206	24.6	4.75	0.03
Atrial fibrillation	97	10.1	14	11.6	83	9.9	0.32	0.57
Coronary Artery Disease	183	19.1	28	23.1	155	18.5	1.46	0.23
Stroke	74	7.7	11	9.1	63	7.5	0.36	0.55
Inflammatory bowel disease	6	0.6	0	0.0	6	0.7	0.87	0.35
Renal failure	66	6.9	10	8.3	56	6.7	0.41	0.52
Liver cirrhosis	20	2.1	1	0.8	19	2.3	1.08	0.30
At-home O_2_ therapy	215	22.4	20	16.5	195	23.3	2.78	0.10
Oral anticoagulation	89	9.3	9	7.4	80	9.6	0.56	0.45
Antiplatelet therapy	347	36.2	49	40.5	298	35.6	1.10	0.30
purulent sputum	230	24.0	20	16.5	210	25.1	4.27	0.04
Clinical signs of deep venous thrombosis	81	8.5	30	24.8	51	6.1	47.76	<0.001
SpO_2_ < 90 mmHg or O_2_ therapy	535^&^	58.0	70	60.9	465	57.5	0.23	0.63
At least one abnormal ECG indicates pulmonary embolism	187^&&^	19.6	32	26.4	155	18.7	4.23	0.04
pCO_2_ < 40 mmHg	414^&&&^	48.2	66	60.0	348	46.5	8.35	0.004
pH > 7.45	255^&&&^	29.7	38	34.5	217	29.0	1.43	0.23
Normal chest X-ray	182^&&&&^	21.1	36	35.0	146	19.3	13.39	<0.001

^&^Data of 35 patients were lost (Patients with PE: 6; Patients without PE: 29); ^&&^Data of 6 patients were lost (Patients without PE: 6); ^&&&^Data of 99 patients were lost (Patients with PE: 11; Patients without PE: 88); ^&&&&^Data of 97 patients were lost (Patients with PE: 18; Patients without PE: 79).

### 3.2 Analysis of clinical and pathological features of patients

In this study, a total of 778 patients (81.2%) had at least one disease, while 220 patients (23.0%) had two or more concurrent diseases. During hospitalization, 887 cases (92.6%) underwent chest X-rays, with 181 cases (20.4%) showing no significant changes, 323 cases (36.4%) exhibiting pulmonary infiltrative changes, and 130 cases (14.7%) presenting pleural effusion. Arterial blood gas analysis was conducted in 870 cases (90.8%), revealing that 256 cases (29.4%) had a pH value ≥7.45, and 415 cases (47.7%) had PaCO_2_ levels within or below the normal range. The average duration of hospitalization for patients was (14.4 ± 8.5) days. Among them, 50 cases (5.18%) resulted in death during hospitalization, including 7 cases (5.8%) of patients with PE. Compression ultrasound was performed in 164 cases (17.1%), and 62 cases (6.42%) were found to have complications of DVT. Interestingly, among the patients without PE on chest vascular CT, 26 individuals were diagnosed with DVT, accounting for 2.71% of the total population and 41.93% of the DVT patients.

### 3.3 Comparison of laboratory test indexes of patients

In comparison to the control group, the observation group exhibited considerably higher levels of RDW-SD and RDW-CV (*p* < 0.001). The albumin (ALB) level in the observation group was markedly lower than that in the control group (*p* < 0.001). Furthermore, the observation group displayed significantly elevated levels of ALT, AST, and lactate dehydrogenase (LDH-L) in contrast to the control group (*p* < 0.001). Additionally, the D-dimer concentration in the observation group was notably higher than that in the control group (*p* < 0.001).

The levels of RBC, Hb, Hct, PLT, platelet crit (PCT %), eosinophils (EO %), lymphocytes (LYM %), oxygen saturation (SO_2_%), partial pressure of oxygen (PO_2_%), and fibrinogen (FIB) in the observation group exhibited significant reduction compared to the control group (*p* < 0.05). Furthermore, the observation group demonstrated significantly elevated MPV, neutrophils (Neut %), lactate (Lac %), and prothrombin time (PT) in comparison to the control group (*p* < 0.05). Table 2 introduces a comparative analysis of laboratory test parameters, encompassing blood count, biochemical measures, blood gas analysis, and coagulation markers, between the two patient groups.

**Table 2:** Comparison of laboratory test indexes included in patients.

Parameters	COPD with PE	COPD without PE	*P* value
WBC (×10^9^/l)	8.41 (5.69–10.87)	8.26 (6.3–10.31)	0.961
RBC (×10^12^/l)	4.18 ± 0.67	4.50 ± 0.57	0.031
HB (g/l)	124.89 ± 17.50	135.07 ± 18.12	0.006
HCT (%)	37.5 (35.05–42.05)	41.5 (37.9–45.3)	0.013
MCV (fl)	92.78 ± 7.13	92.66 ± 5.04	0.789
MCH (Pg)	29.87 ± 2.50	30.14 ± 1.73	0.769
MCHC (g/l)	321.60 ± 15.91	321.55 ± 10.70	0.894
PLT (×10^9^/l)	162.9 (131.6–247)	209 (175–256)	0.010
RDW-SD (fl)	48.8 (43.7–52.37)	43.9 (41.0–45.2)	< 0.001
RDW-CV (%)	14.8 (12.78–15.37)	12.8 (12.0–13.9)	< 0.001
PDW (fl)	12.05 (11.0, 13.8)	11.3 (9.6–13.4)	0.059
MPV (fl)	10.66 ± 1.41	9.67 ± 1.09	0.025
PCT (%)	0.19 (0.14–0.28)	0.22 (0.19–0.26)	0.045
P-LCR (%)	28.79 ± 9.49	25.60 ± 8.11	0.062
EO (×10^9^/l)	0.03 (0.01–0.09)	0.08 (0.02–0.19)	0.001
NEUT (%)	79.4 (75.26–85.21)	75.1 (69.4–83.4)	0.015
LYM (%)	12.44 (8.14–15.44)	15.69 (10.0–20.9)	0.035
MONO (%)	6.39 (4.89–8.31)	6.5 (5.3–8.3)	0.370
EO (%)	0.64 (0–1.14)	1.1 (0.4–2.5)	0.010
ALB (g/l)	35.5 (33.8–38.8)	38.9 (35.0–41.4)	< 0.001
ALT (μ/l)	24 (13–33.7)	14 (9–19)	< 0.001
AST (*μ*/l)	18.4 (14.4–30.4)	17 (12–18)	< 0.001
LDH-L (μ/l)	238.0 (194.0–277.0)	176 (155–202)	< 0.001
CHOL (mmol/l)	4.42 (3.51–4.75)	4.21 (3.71–5.2)	0.394
TG (mmol/l)	1.34 (0.96–1.56)	1.16 (0.78–1.48)	0.065
CREA (μmol/l)	59.0 (51–87.34)	58.6 (54–74)	0.932
UA (μmol/l)	238.2 (172–374.90)	245 (211–327.6)	0.879
PH	7.46 (7.5–7.57)	7.53 (7.51–7.56)	0.328
SO_2_ (%)	92.4 (88.54–96.34)	95.69 (93.38–97.4)	0.003
PO_2_ (mmHg)	64.36 (52.9–77.2)	73.3 (64.4–75.1)	0.005
PCO_2_ (mmHg)	42.3 (35.9–52.5)	43.5 (41–50.9)	0.343
Lac (mmol/l)	1.76 (1.34–2.34)	1.56 (1.1–1.95)	0.033
PT (sec)	13.9 (13.0, 15.2)	13.1 (12.8, 14.0)	0.009
APTT (sec)	36.9 (31.1–41.9)	36.2 (32.6–38.6)	0.811
TT (sec)	16.8 (15.54–19.65)	16.6 (15.7–17.5)	0.316
FIB (g/l)	3.65 (2.32–5.17)	4.74 (3.5–5.95)	0.002
D-Dimer (μg/ml)	2.61 (1.86–9.14)	0.43 (0.30–0.73)	< 0.001

### 3.4 Univariate and multivariate analysis of clinical and pathological risk factors associated with the prevalence of PE

A higher occurrence of PE was found to be significantly linked with various factors based on the univariate analysis. These factors encompassed age, female gender, previous occurrence of venous thromboembolism, the existence of at least one ECG abnormality implying PE, pH ≥ 7.45, PaCO_2_ ≤ 40 mm Hg, absence of anomalies in a chest radiograph, clinical indications and symptoms indicative of deep vein thrombosis, recent surgical procedure, recently extended bed rest for at least 3 days, and clinical records of heart failure and hypertension (*p* < 0.05). Conversely, patients with malignant tumors and those with purulent sputum had a significantly lower prevalence of PE. The summarized results of the univariate analysis can be found in Table 3.

**Table 3:** Univariate and multivariate analysis of risk factors of clinical characteristics.

	Univariate		Multivariate	
	Adjusted OR	95% CI	Adjusted OR	95% CI
Age (Mean ± SD, years)	1.06	1.03;1.08#	1.04	1.02;1.07#
Sex - Men	0.50	0.35;0.71#	0.41	0.27;0.64#
Recent bed rest ≥3 days	1.68	1.13;2.47*	Not selected	Not selected
Recent surgery	2.08	0.91;4.72*	Not selected	Not selected
Previous venous thromboembolism	2.30	1.36;3.88#	Not selected	Not selected
Malignant tumor	0.61	0.37;1.02	–	–
Heart failure	1.43	0.84;2.45*	Not selected	Not selected
Diabetes mellitus	0.80	0.49;1.33	–	–
Hypertension	1.55	1.03;2.30*	1.61	1.02;2.62*
Atrial fibrillation	1.20	0.69;2.12	–	–
Coronary artery disease	1.33	0.85;2.06	–	–
Stroke	1.20	0.62;2.30	–	–
Renal failure	1.28	0.66;2.49	–	–
Liver cirrhosis	0.34	0.06;2.45	–	–
At-home O_2_ therapy	0.65	0.40;1.05	–	–
Oral anticoagulation	0.80	0.41;1.56	–	–
Antiplatelet therapy	1.20	0.82;1.75	–	–
Purulent sputum	0.58	0.36;0.95	–	–
Clinical signs of deep venous thrombosis	5.20	3.22;8.39#	5.64	3.13;10.16#
SpO_2_ < 90 mmHg or O_2_ therapy	1.14	0.77;1.69	–	–
At least one abnormal ECG indicates pulmonary embolism	1.55	1.01;2.38*	Not selected	Not selected
pCO_2_ < 40 mmHg	1.74	1.18;2.56#	1.98	1.25;3.15#
pH > 7.45	1.24	0.82;1.88*	Not selected	Not selected
Normal chest X-ray	2.22	1.43;3.41#	1.91	1.15;3.14#

*p < 0.05; #*p* < 0.01.

In the analysis that accounts for multiple variables, numerous factors were discovered to exhibit a significant correlation with a greater occurrence of PE (*p* < 0.05). These factors encompass age, being female, the presence of clinical indications implying deep vein thrombosis, high blood pressure, PaCO_2_ ≤ 40 mmHg, and a chest X-ray within normal limits. Additional information can be seen in Table 3.

Patients in the observation group experienced a slightly longer average hospital stay in comparison to those in the control group (15.8 days versus 14.2 days, *p* = 0.06). Despite a slightly higher in-hospital mortality rate being observed in the observation group, this difference did not reach statistical significance (5.8% versus 5.1%, *p* = 0.76).

### 3.5 Logistic regression analysis of laboratory indexes of patients

The laboratory parameters exhibiting significant disparities (*p* < 0.001), as shown in Table 2, were integrated into the multifactor logistic regression model. Assessing the logistic regression model in Table 4 unveils the autonomous factors positing risks for PE. LDH-L, RDW-SD, and D-dimer were identified as autonomous risk factors for PE in elderly COPD patients (*p* < 0.05).

**Table 4:** Logistic regression model analysis showed independent laboratory index risk factors of PE.

Variable	Adjusted OR	95% CI	*p-*value
RDW-SD (fl)	1.179	1.037–1.359	0.005
RDW-CV (%)	1.024	0.847–1.186	0.793
ALB (g/l)	0.925	0.763–1.018	0.149
ALT (μ/l)	1.007	0.976–1.043	0.626
AST (μ/l)	0.973	0.910–1.063	0.711
LDH-L (μ/l)	1.024	1.021–1.046	0.029
D-Dimer (μg/ml)	1.515	1.041–2.204	0.025

### 3.6 Analysis of the diagnostic value of RDW and D-dimer in predicting PE in patients with COPD

**Figure 2:**
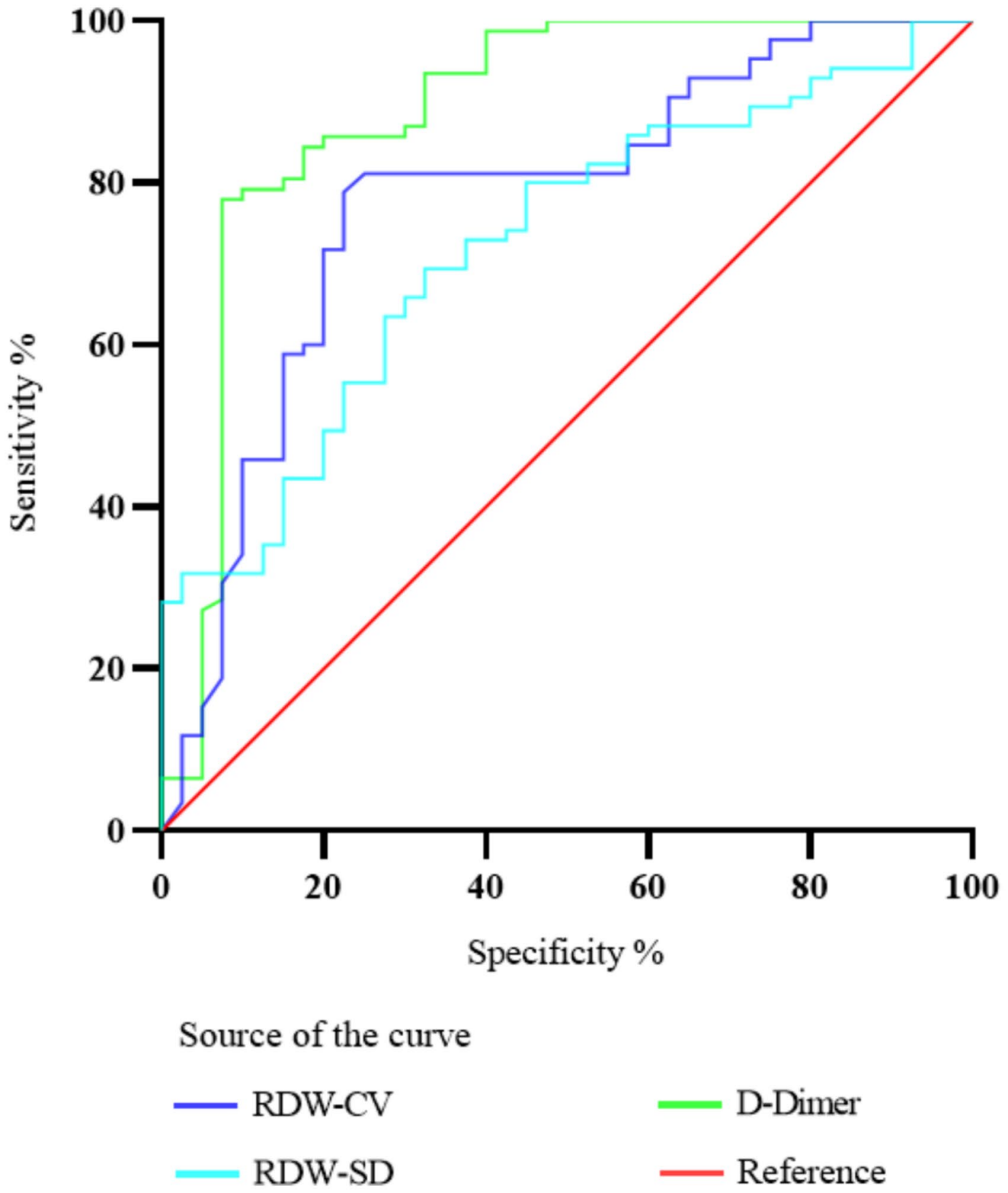
Diagnostic value of ROC curve comparison of RDW-CV, RDW-SD, and D-dimer in predicting PE in patients with COPD.

To assess the predictive precision of RDW-CV, RDW-SD, and D-dimer for PE, ROC curve analysis was conducted. The resulting AUC values were determined as 0.778, 0.723, and 0.887, correspondingly. The critical values that yielded the best prediction for PE were determined to be 46.25 for RDW-SD, 1.747 for D-dimer, and 13.59 for RDW-CV ratio. The corresponding sensitivity and specificity values were 75.59 and 67.5% for RDW-SD, 82.92 and 92.5% for D-dimer, and 78.82 and 77.5% for RDW-CV ratio. Please refer to Figure 2 for a visual representation of the results. Table 5 provides detailed statistics on the prediction accuracy of RDW and D-dimer.

**Table 5:** Comparison of discriminant ability between RDW and D-dimer in predicting COPD and PE.

Parameters	RDW-CV	RDW-SD	D-Dimer
Cut off	13.59	46.25	1.747
AUC	0.778	0.723	0.887
95% CI	0.6863 to 0.8687	0.6312 to 0.8141	0.8123 to 0.9621
Sensitivity (%)	78.82	75.59	82.92
Specificity (%)	77.5	67.5	92.5
*p*-value	<0.001	<0.001	<0.001

## 4 Discussion

During this investigation, a noteworthy connection emerged between the prevalence of PE and the clinical indicators and manifestations of deep vein thrombosis (DVT) (with an odds ratio of around 5.6). Merely a minor proportion of individuals (6.1%) did not encounter the development of PE. From a pathophysiological perspective, it has been suggested that patients with COPD and PE may have reduced partial pressure of carbon dioxide (25). However, there is ongoing debate regarding the clinical evidence supporting this view. Some studies have reported that PE may occur due to a decrease in PaCO_2_ during COPD exacerbations, while others have argued that the diagnostic value of arterial blood gas testing in PE is limited unless included in the clinical score (26, 27). In our investigation, we veer towards the latter perspective since we noted a reduction in the carbon dioxide’s partial pressure in 60.0% of individuals identified with PE and in 46.5% of those without a diagnosis of PE.

Earlier research has shown that there is a high occurrence of imaging alterations, specifically consolidation on chest radiographs, among senior individuals diagnosed with COPD (28). Furthermore, these changes have been associated with an unfavorable prognosis. According to our research findings, around 21.1% of individuals diagnosed with COPD exhibited normal results in their chest X-rays. This observation was linked to a higher probability of developing VTE, with an odds ratio of approximately 1.9. Furthermore, our study identified that among elderly COPD patients who do not experience PE, the presence of other risk factors alongside “clinical indications and manifestations of DVT” does not serve as an efficient method for diagnosing or excluding the possibility of PE.

According to this study, it appears that PE has a limited impact on patient prognosis. In the case of elderly individuals diagnosed with COPD, there is no mortality disparity of statistical significance observed when comparing patients with and without PE (5.8% vs. 5.1%, *p* = 0.76). Additionally, the hospital stay for patients with PE was only slightly longer compared to those without PE (15.8 days vs. 14.2 days, *p* = 0.06). This suggests that hospitalization during diagnosis and subsequent treatment effectively reduces the risk of death and lengthier hospital stays.

The investigation revealed that there was a noticeable increase in the levels of RDW-CV and RDW-SD among elderly COPD patients with PE in comparison to those who did not have PE. Moreover, heightened RDW-SD levels exhibited a considerably augmented likelihood of PE, whereas diminished RDW-SD levels were linked to a decreased risk. The escalated susceptibility to PE in COPD patients might be attributed to multiple mechanisms, encompassing systemic inflammation (29), hypoxemia (30), amplified oxidative stress (31), dysfunction of endothelium (32), and an inclination towards increased blood clot formation (33). The results derived from this investigation imply that RDW has the potential to function as an alternative biomarker to gauge the level of inflammatory activity. Previous studies have also indicated a possible link between inflammation and chronic thromboembolic pulmonary hypertension in RDW (34). The occurrence of these irregularities can involve the amplified release of erythropoietin and an undetected sudden decline in cardiac functionality.

A multivariate analysis employing logistic regression was carried out to assess the influence of various risk factors on the presence of both COPD and PE. Our study reveals that RDW-SD, LDH-L, and D-dimer possess distinct predictive significance for PE, regardless of the presence of other risk factors. The risk of PE is significantly increased by both elevated and decreased levels of D-dimer. Additionally, an augmented risk of PE is closely linked with RDW-SD, underscoring its involvement as a contributing factor to PE. Detecting PE early is of utmost importance for patients at high risk, and doctors can assess the risk in COPD and PE patients by considering the levels of RDW-SD.

RDW, an easily measured laboratory factor that is relatively simple, inexpensive, and has been recently demonstrated to predict mortality and morbidity in various diseases, has emerged as a potential predictor of PE prognosis (35). In one study involving 702 PE patients, RDW levels were found to be a potential marker for mortality (36). Previous research has indicated that blood RDW levels have the potential to serve as an uncomplicated and convenient measure for forecasting the likelihood of mortality within 30 days for individuals with PE. In addition, Hammons et al. noted a connection between RDW and prognosis, severity, and survival rates in patients experiencing acute PE (37).

Nevertheless, limited investigations have focused on the potential of RDW in predicting PE specifically in COPD patients. Analysis of ROC curves has demonstrated that D-dimer exhibits a higher AUC in predicting PE compared to RDW-SD. Notably, while D-dimer demonstrates superior diagnostic accuracy in acute PE detection, our findings highlight RDW’s unique clinical merits as a chronic disease progression marker. The persistent elevation of RDW may reflect cumulative inflammatory burden and hypoxia-mediated erythrocyte dysfunction in COPD patients (30, 34), potentially serving as an early warning sign for subclinical thrombotic predisposition prior to acute events. Particularly in scenarios where D-dimer interpretation is confounded by recent surgery, trauma, or chronic inflammatory states (20), RDW provides complementary value through its stability across these clinical conditions. Future investigations should explore optimized biomarker panels combining RDW’s chronic risk stratification capacity with D-dimer’s acute phase responsiveness, potentially enhancing PE prediction in complex COPD populations. This dual-marker approach could prove crucial for implementing personalized anticoagulation strategies in elderly COPD patients with varying thrombotic risk trajectories. As a result, we propose the hypothesis that heightened levels of RDW could potentially function as a reliable marker for the presence of PE in COPD patients.

## 5 Conclusion

In this multicenter retrospective research, we discovered that 2.71% of elderly patients with COPD were diagnosed with DVT without PE. This finding highlights the importance of considering the prevalence of VTE. Using multivariate analysis, we discovered numerous potential clinical risk factors associated with PE in patients with COPD. These factors include being female, exhibiting clinical symptoms and signs indicating DVT, having hypertension, having a PaCO2 level below 40 mmHg, and presenting with a normal chest X-ray. Moreover, we uncovered that RDW-SD might serve as a promising biomarker for the diagnosis of PE in elderly individuals diagnosed with COPD.

## Data Availability

The original contributions presented in the study are included in the article/Supplementary material, further inquiries can be directed to the corresponding authors.

## Glossary

COPD: chronic obstructive pulmonary disease
PE: pulmonary embolism
RDW: red cell distribution width
DVT: deep venous thrombosis
CTPA: CT pulmonary angiography
APTT: activated partial thromboplastin time
PT: prothrombin time
WBC: white blood cell
RB: red blood cell
Hb: hemoglobin
PLT: platelet count
RDW-CV: coefficient of variation
RDW-SD: standard deviation
MPV: mean platelet volume
PDW: platelet distribution width
Hct: hematocrit
MCV: mean corpuscular volume
MCH: mean corpuscular hemoglobin
MCHC: mean corpuscular hemoglobin concentration
PCT: platelet crit
EO: eosinophils
LYM: lymphocytes
MONO: monocytes
Neut: neutrophils
ALB: albumin
ALT: alanine aminotransferase
AST: aspartate aminotransferase
LDH-L: lactate dehydrogenase
CHOL: cholesterol
TG: triglyceride
CREA: creatinine
UA: uric acid
SO_2_: oxygen saturation
PO_2_: partial pressure of oxygen
PaCO_2_: partial pressure of carbon dioxide
Lac: lactate
TT: thrombin time
FIB: fibrinogen
ROC: receiver operating characteristic
AUC: area under the curve
CI: confidence interval
OR: odds ratio
VTE: venous thromboembolism
HIF-2: hypoxia-inducible factor-2
AECOPD: acute exacerbation of chronic obstructive pulmonary disease
ESC: European society of cardiology
